# Physiological genetic variation in tomato fruit chilling tolerance during postharvest storage

**DOI:** 10.3389/fpls.2022.991983

**Published:** 2022-09-08

**Authors:** Sivan David, Elena Levin, Elazar Fallik, Sharon Alkalai-Tuvia, Majid R. Foolad, Amnon Lers

**Affiliations:** ^1^Department of Postharvest Science, Volcani Institute, Agricultural Research Organization, Rishon LeZion, Israel; ^2^Robert H. Smith Faculty of Agriculture Food and Environment, The Robert H. Smith Institute of Plant Sciences and Genetics in Agriculture, The Hebrew University of Jerusalem, Rehovot, Israel; ^3^Department of Plant Science, The Pennsylvania State University, University Park, PA, United States

**Keywords:** postharvest chilling tolerance, cold storage, antioxidant potential, chlorophyll fluorescence, *Solanum lycopersicum*, *Solanum pimpinellifolium*

## Abstract

Storage at low temperatures is a common practice to prolong postharvest life of fruit and vegetables with a minimal negative impact on human/environmental health. Storage at low temperatures, however, can be restricted due to produce susceptibility to non-freezing chilling temperatures, when injuries such as physiological disorders and decays may result in unmarketable produce. We have investigated tomato fruit response to postharvest chilling stress in a recombinant inbred line (RIL) population developed from a cross between a chilling-sensitive cultivated tomato (*Solanum lycopersicum* L.) breeding line and a chilling-tolerant inbred accession of the tomato wild species *S. pimpinellifolium* L. Screening of the fruit of 148 RILs under cold storage (1.5°C) indicated presence of significant variations in chilling tolerance, manifested by varying degrees of fruit injury. Two extremely contrasting groups of RILs were identified, chilling-tolerant and chilling-sensitive RILs. The RILs in the two groups were further investigated under chilling stress conditions, and several physiological parameters, including weight loss, chlorophyll fluorescence parameters *Fv/Fm*, and *Performance Index* (*PI*), were determined to be efficient markers for identifying response to chilling stress in postharvest fruit. The *Fv/Fm* values reflected the physiological damages endured by the fruit after cold storage, and *PI* was a sensitive marker for early changes in *photosystem II* function. These two parameters were early indicators of chilling response before occurrence of visible chilling injuries. Antioxidant activities and ascorbic acid content were significantly higher in the chilling-tolerant than the chilling-sensitive lines. Further, the expression of C-repeat/DREB binding factors (*CBFs*) genes swiftly changed within 1-hr of fruit exposure to the chilling temperature, and the *SlCBF1* transcript level was generally higher in the chilling-tolerant than chilling-sensitive lines after 2-hr exposure to the low temperature. This research demonstrates the presence of potential genetic variation in fruit chilling tolerance in the tomato RIL population. Further investigation of the RIL population is underway to better understand the genetic, physiological, and biochemical mechanisms involved in postharvest fruit chilling tolerance in tomato.

## Introduction

Postharvest losses of horticultural fresh food produce are estimated to be between 30 and 50% worldwide (Hodges et al., 2011; Buzby and Hyman, 2012; Porat et al., 2018). Fruit and vegetables together account for ~66% of total food losses. Reducing postharvest losses is one of the leading solutions to ensure global food security (Kader, 2005). Cold storage is a common practice to prolong the postharvest performance of crop produce, with a minimal negative impact on human health or the environment (McGlasson et al., 1979). Low (chilling) temperatures, below 10°C but above commodities freezing points, maintain the postharvest quality of fruit and vegetables by reducing respiration and other basic metabolic processes involved in senescence and ripening; hence, deterioration is delayed. Chilling temperatures also slow down the development of pathogenic microorganisms, which otherwise may lead to rapid decay and produce loss. Many vegetable crops, in particular those originated from tropical and subtropical regions, however, are susceptible to chilling temperatures and thus the application of cold storage may be restricted (McGlasson et al., 1979; Sevillano et al., 2009). Crop produce stored at temperatures below their chilling tolerance threshold suffer injuries, including skin pitting, internal or surface browning, water-soaked tissue, and abscission (Wang, 1986; Brummell et al., 2004; Valenzuela et al., 2017). Chilling injury symptoms may also appear as swelling and disorganization of cell organelles, tissue collapse, and ultimately death (Kratsch and Wise, 2000; Sevillano et al., 2009). Further, chilling injuries increase water loss from the fruit due to damages to physical barriers of the pericarp cells, including loss of membrane integrity, tissue collapse, and cracks formation on the fruit skin. Subsequently, the primary chilling injuries may become preferred sites for pathogens, resulting in accelerated decay and massive produce losses. Therefore, chilling sensitive crops may have to be stored at a higher minimum temperature, which would decrease their marketable life. In some cases, it may also necessitate employment of more expensive? storage strategies or using chemicals that would negatively impact human health and the environment (Wang, 2010).

Through natural selection and evolution, some plant species have acquired genetic capabilities for cold tolerance, enabling their success under sub-optimal temperatures (Venema et al., 2005; Knight and Knight, 2012). Such adaptations are through evolution of complex molecular and physiological processes at the cell and whole plant levels, understanding of which may facilitate development of chilling tolerance *via* crop improvement (Hsieh et al., 2002; Zhu et al., 2007; Shi et al., 2018). Cold sensing and plant response to low temperatures involve many different signal transduction pathways, regulatory circuits, and biological processes (Knight and Knight, 2012; Miura and Furumoto, 2013; Zhu, 2016), including the ICE1-CBF/DREB-COR transcriptional networks (Thomashow, 1999). Most previous studies regarding low-temperature sensing and plant response to cold stress were conducted during plant vegetative growth and reproductive stages. It is unknown whether the genetic and physiological mechanisms involved in plant response to cold stress during its development are relevant to fruit exposed to chilling temperatures postharvest. To develop crop plants with the fruit ability to tolerate chilling stress during postharvest storage, it is necessary to study and understand the primary physiological and genetic mechanisms involved in fruit response to chilling temperatures after harvest.

Plants of the cultivated tomato (*Solanum lycopersicum* L.) are generally very sensitive to low temperatures during their growth and development (Van Der Ploeg and Heuvelink, 2005; Venema et al., 2005), and their fruit are also highly sensitive to chilling temperatures during postharvest storage (Thorne and Segurajauregui, 1982; Hobson, 1987; Gomez et al., 2009). Harvested tomato fruit may develop chilling injuries (CI) when stored in temperatures below 10°C. Similar to that for many other traits, genetic variation in cold tolerance was most likely lost in the cultivated tomato through its domestication and early breeding (Venema et al., 2005). Such variation, however, has been reported in some wild tomato accessions, in particular accessions originated from high elevations in South America (Wolf et al., 1986; Foolad and Lin, 2001a; Venema et al., 2005). To develop tomato cultivars with cold/chilling tolerance, genetic and physiological characterization of wild tomato accessions with such desirable attributes is necessary. We have established a comprehensive collaborative research program between Israel and the United States to identify and characterize sources of cold/chilling tolerance in tomato and develop new germplasm with improved tolerance, including fruit chilling tolerance during postharvest storage.

In the present study, we have employed a recombinant inbred line (RIL) population, previously developed at Penn State University from a cross between *Solanum pimpinellifolium* accession LA2093 and tomato breeding line NC EBR1 (Ashrafi et al., 2009), to investigate the genetic and physiological basis of postharvest fruit chilling tolerance in tomato. It should be noted that, the use of RILs for genetic and physiological studies can be advantageous due to several reasons, including (1) presence of high level of homozygosity and thus the ability to regenerate the population without changing its genetic make-up, (2) the opportunity to repeat experiments in time or space and under different environmental conditions (e.g., field vs. greenhouse), (3) accurate separation and estimation of genetic and environmental effects on trait expression, (4) increasing trait heritability by reducing environmental variation *via* repeating experiments, (5) reliable gene/QTL mapping for traits segregating in the population, and (6) comparatively uniform genetic backgrounds among the RILs minimizing genetic noises. The latter feature, for example, facilitates reliable identification of genes or physiological mechanisms underlying expression of a particular trait such as post-harvest chilling tolerance. In general, for specific traits, such as chilling tolerance, results obtained by using RILs (or near-isogenic lines; NILs) are more reliable than those obtained by using genotypes with contrasting genetics for many other traits. Previously, this RIL population was used in numerous studies, including investigation of genetic controls of fruit quality traits (Ashrafi et al., 2012; Kinkade and Foolad, 2013) and disease resistance (Ashrafi and Foolad, 2015). Further, recently we developed a highly-saturated genetic linkage map of this RIL population based on more than 140,000 SNP markers (Gonda et al., 2019), which facilitates employment of this population for in depth studies.

In this study, we have determined that the fruit of LA 2093 exhibits chilling tolerance during postharvest storage, and that there is significant variation among the RILs in fruit chilling tolerance. We also report our findings on the characterization of tomato fruit chilling tolerance in this RIL population.

## Materials and methods

### Plant material

A tomato recombinant inbred line (RIL) population (*n* = 148 lines), previously developed at the Penn State University from an interspecific cross between *Solanum pimpinellifolium* accession LA2093 (staminate parent) and S. *lycopersicum* breeding line NC EBR1 (Ashrafi et al., 2009), was used in this study. Accession LA 2093 is a red-fruited inbred line with indeterminate growth habit and numerous desirable characteristics, including high fruit quality, disease resistance, and tolerance to abiotic stresses (Ashrafi et al., 2009). NC EBR1 is an advanced fresh-market tomato breeding line with numerous desirable horticultural characteristics, developed at NC State University (Gardner, 1988). Further, prior to the examination of the RIL population for chilling tolerance/sensitivity during postharvest storage at low temperatures, we determined that fruit of LA 2093 exhibited chilling tolerance and fruit of NC EBR1 exhibited chilling sensitivity during postharvest storage (described below). Original seeds of LA 2093 and NC EBR1 were received from the CM Rick Tomato Genetic Resource Center (TGRC), California, USA (http://tgrc.ucdavis.edu/), and RG Gardner, NC State University, North Carolina, USA (https://mountainhort.ces.ncsu.edu/fresh-market-tomato-breeding/rggardner/), respectively. In the present study, the RIL population and its two parental lines were used for experiments on fruit chilling tolerance during postharvest storage, as described below.

### Growth of the RIL population and production of fruit for chilling treatment

Eight to 10 plants each of the 148 RILs and parental lines were grown in an open field at the Faculty of Agriculture Experimental Station in Rehovot, Israel, during April-August. At mature green (MG) stage, fruit were harvested in the morning (between 6 and 9 am) every 2 weeks. Fruit were harvested from at least 3 plants for each of the RILs and parental lines. For each line, efforts were made to harvest fruit of similar size, shape and maturity, and fruit calyxes were removed immediately after harvest. Fruits were washed in 0.03% sodium hypochlorite (NaClO, liquid bleach), rinsed with tap water, dried, and divided randomly for the different experiments (described below). In addition to the field-grown plants, 22 selected RILs (described below) were grown in pots containing artificial soil (Green 77 artificial soil, Even-Ari Ltd., Beit Elazari, Israel) in a greenhouse (GH) at the Volcani Institute, Rishon LeZion, Israel, under daily temperature of ~25–30°C (and natural light) and night temperature of ~17–23°C. Fruit from the GH-grown plants were prepared for experiments similar to those from the field-grown plants.

### Postharvest cold storage treatment and chilling injury evaluation

Mature-green fruit of the two parental lines and 148 RILs were placed in paper bags and stored in cold storage room, set at 1.5 ± 0.5°C and 90 −97% RH, for 5 or 14 days. For the control (non-chilling) treatment, fruit were stored at 12°C with 90–97% RH for 14 days. Chilling injury symptoms were evaluated after 14 days at 1.5°C, followed by evaluation after 3-d incubation at 20°C and 65–70% RH to allow any tissue damage to appear. To quantify damages occurred on the fruit, a chilling injury (CI) visual index was used (described below). Chilling injuries were generally manifested as surface pitting, glazing, scalding, tissue decomposition, softening, uneven ripening, and subsequent growing of pathogens. The severity of chilling injuries (CI) was assessed visually, mainly based on the development of surface pitting and tissue injuries, on a scale of 0–4, where 0 = no damage; 1 = light damage, covering <10% of the fruit surface; 2 = medium damage, covering 10–30% of the fruit surface; 3 = severe damage, covering 40–60% of the fruit surface; and 4 = very severe damage, covering more than 70% of the fruit surface. The CI index was calculated as follows:


$$\begin{array}{l} \text{CI index}=\Sigma \;\left(\text{CI level}\;*\;\text{number of fruit at the CI level}\right)/\\ \text{ total number of fruit in the treatment} \end{array}$$

Examples for the observed chilling injuries of the fruit surface are shown in Supplementary Figure 1.

### Weight loss measurements

Chilling injuries are expected to increase water loss from the fruit due to damages to physical barriers of the pericarp cells, including loss of membrane integrity, tissue collapse, and cracks formation on the fruit skin. Fruit water loss due to cold storage was examined in 5 “chilling tolerant” and 6 “chilling sensitive” RILs. To measure water loss, fruit weight (FW) was measured at different stages, including immediately after harvest (before storage), at the end of the 14-day cold storage, and after additional 3 days storage at 20°C. In parallel, fruit were also stored for 14 days at an optimal low temperature of 12°C as a control for low but non-chilling temperature. For each RIL, at least 12 fruit from 3 different plants were sampled for FW measurement, and all measurements were repeated in 3 different harvests. Fruit water loss was measured as the percentage reduction in FW compared to the initial FW.

### Trolox equivalent antioxidant capacity assay

To evaluate total antioxidant activity in tomato samples, the ABTS^•+^ radical decolorization assay was employed (Miller and RiceEvans, 1997; Goldenberg et al., 2014). The ABTS assay measures the relative ability of antioxidants to scavenge the ABTS radicle. Frozen fruit samples (200 mg each sample) were ground to powder in LN, and samples were extracted with 1 mL of 0.2 M acetate buffer (pH = 4.3) followed by centrifugation at 13,000 *g* for 15 min at 4°C. For each sample, the supernatant was transferred to a new tube and centrifuged again for additional 5 min. The reaction mixture contained 1 mL of 150 μM 2,2'-azinobis-(3-ethylbenzthiazoline-6-sulphonic acid ABTS^•+^) and 75 μM K_2_O_8_S_2_ in acetate buffer, pH = 4.3 and 10 μL sample of the supernatant tomato extract. The mixture was incubated in the dark for 15 min at room temperature, and subsequently the absorbance of the mixture was measured at 734 nm to determine the Trolox Equivalent (T.E.) antioxidant activity, as indicated by the degree of disappearance of the blue color. The blank (control) used for this experiment was 0.2 M acetate buffer (pH = 4.3), and the standard samples were prepared with 10 μL of 1 mM Trolox and 1 mL ABTS^+^ regent. The T.E. was calculated, as described elsewhere (Goldenberg et al., 2014).

### Ascorbic acid measurement

Ascorbic acid (AsA) measurements were performed using the Folin reagent method (Jagota and Dani, 1982). Fresh fruit tissue (200 mg) was ground and mixed with 0.8 mL 10% (w/v) TCA and centrifuged for 20 min at 12,000 X g. The supernatant was transferred to a new tube. For the reaction mixture, 1.6 mL ddH2O and 0.2 mL of 10 fold diluted Folin reagent (F9252, Sigma Aldrich, Louis, MO, U.S.) were mixed with 0.4 mL fruit extract. The reaction mixture was incubated at room temperature for 10 min, and absorbance at 760 nm was measured by a spectrophotometer. The concentration of AsA was calculated according to a standard calibration curve prepared from commercial AsA (F320927, Merck, Darmstadt, F.R, DE). Three to six biological replicates were taken for each tomato RIL for each experiment. Each biological repeat consisted of tissue collected from 3 to 5 fruit.

### Chlorophyll fluorescence parameters

Two chlorophyll fluorescence parameters, *Fv/Fm* and *Performance Index* (*P. Index*), which are generally used to examine plant response to abiotic stresses (Lurie et al., 1994; Baker and Rosenqvist, 2004; Zivcak et al., 2008; Kalaji et al., 2011; Su et al., 2015), were used to evaluate the response of tomato fruit to postharvest chilling stress. The *P. Index* quantifies the overall functionality of the electron flow through the PSII, and is suggested as a sensitive parameter of plant homeostasis (Ceusters et al., 2019). The two parameters were measured on the blossom end of MG fruit, using a portable Handy PEA fluorimeter (Hansatech instruments Ltd, Pentney, UK). Prior to the analysis, tomato fruit were kept in dark for 8 min at room temperature. The instrument sensor includes three ultra-bright red LEDs, producing a peak of 650 nm wavelength at a maximum intensity of 3,500 μmole m^−2^ s^−1^. The duration of illumination on the surface of fruit peel was set to 40 s, after which *Fv/Fm* and *P. Index* were immediately recorded. For each RIL, a total of 12 fruit (harvested from 3 plants) were examined. The measurements were repeated for three different harvests (three experiments).

### Proline content measurement

One g fresh fruit tissue was frozen, ground to power in LN, extracted with 3% (w/v in ddH2O) sulfsalicyic acid, spun in a centrifuge at 12,000 x g, and the supernatant was used to measure proline content in μM/g FW, using a method described elsewhere (Claussen, 2005).

### Quantitative real-time PCR

Mesocarp tissue samples taken from fruit equator area were frozen in LN and kept in falcon tubes at −80°C for later analyses. Total RNA was extracted from each sample using the *Spectrum*™ Total RNA kit (Sigma Aldrich, Louis, MO, USA). First-strand cDNA was synthesized from the RNA, using the *MAXIMA*^*TM*^ cDNA kit (Thermo Scientific, Waltham MA USA), and stored at −20°C for later analyses. The qPCR reactions were performed using a *StepOne*® Real-Time PCR System (Applied Biosystems) and Fast SYBR^TM^ Green Master Mix (Applied Biosystems, cat no. 4385616) and gene-specific primers. Primer sequences were designed using the primer-designing tool Primer-BLAST (NCBI), and the primers' compatibilities were evaluated by NetPrimer software (PREMIER Biosoft International). The expression data were analyzed by *Ct* value normalized to the control gene *UBI3* and quantified by the Delta-Delta cycle threshold method (Livak and Schmittgen, 2001). All experiments were carried out with a non-template control, and repeated for three biological experiments. The list of primers used in the qPCR analysis is shown in Supplementary Table 1.

### Statistical analysis

All data were iterated three times and expressed as the mean ± standard error. Data comparison from the different groups was conducted by one-way ANOVA followed by followed by Tucky HSD tests at *P* < 0.05 using JMP Pro 13.0 software (JMP Inc., Rishon LeZion, IL).

## Results

### Assessment of fruit chilling injury and chilling tolerance

**Figure 1:**
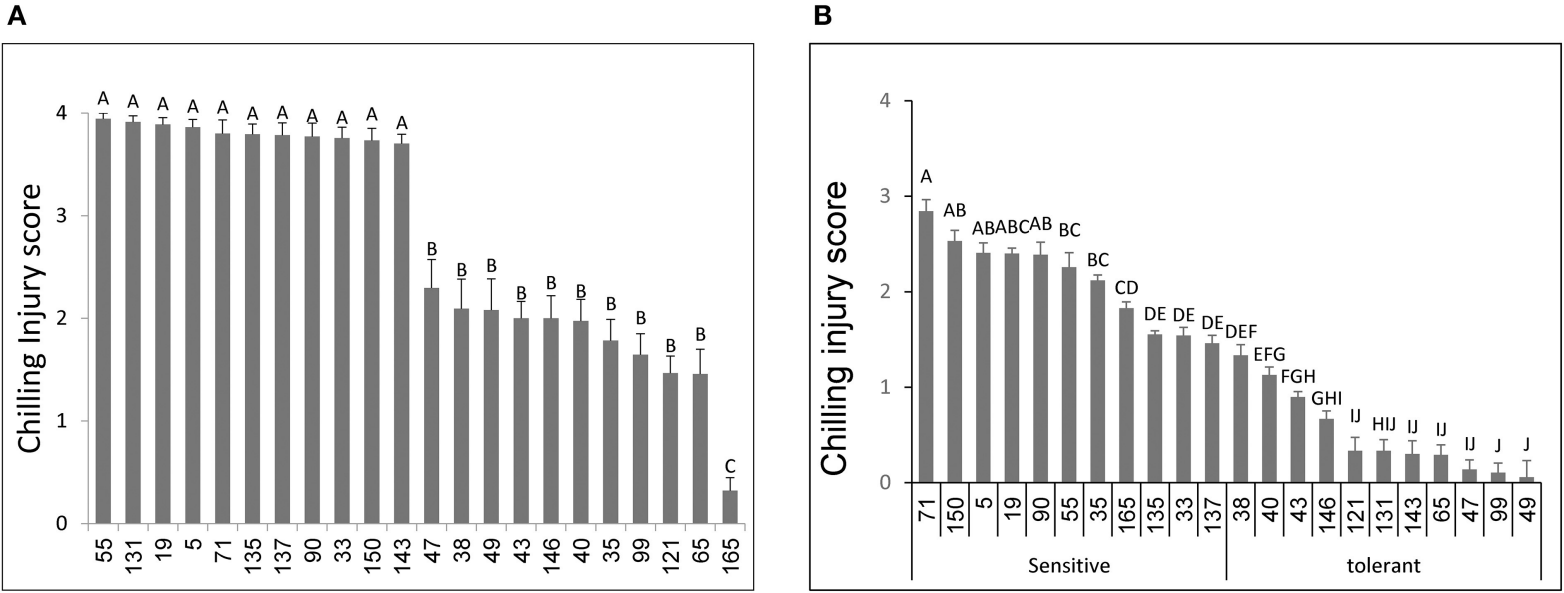
Susceptibility to chilling temperature storage of fruits from different tomato RILs. Fruits at the MG ripening stage were harvested and immediately stored for 14 days at 1.5°C followed by 3 days at 20°C. Chilling injury scores were determined based on visual symptoms as described in Materials and Methods, an average of 30-40 fruits were used for each RIL, harvested at four different dates. Shown here are the results of chilling injuries of tomatoes grown in the field **(A)** and in the greenhouse **(B)**. Vertical bars are standard error means and different letters indicate a significant difference (*p* < 0.05).

Fruit of the *S. lycopersicum* parental line NC EBR1 showed more skin pitting and necrotic lesion and thus significantly higher CI, than fruit of the *S. pimpinellifolium* parent LA2093 (Supplementary Figure 2). Based on the 4 different harvests (4 experiments) from the field-grown plants, there were significant differences in CI among the 148 RILs examined, ranging from low CI lines (similar to LA 2093) to high CI lines (similar to NC EBR-1) (Figure 1A). Based on the initial evaluation of the 148 RILs, we selected 22 with extreme responses: 11 RILs with the lowest CI (~2.0) and 11 RILs with the highest CI (~4.0) (Figure 1A). These selected RILs were grown in a GH, and their MG fruit were harvested and stored under the chilling conditions, as described in M&M. Responses of the selected RILs were generally similar to their responses when fruit were harvested from the field, with a few exceptions (Figure 1B). For example, for RILs 35 and 165, while their field fruit showed some chilling tolerance, their GH fruit exhibited cold sensitivity. Similar, though in opposite direction, results were also observed for RILs 131 and 143. These four RILs, therefore, were eliminated from any further analysis. It should also be noted that, there were some scaling differences between the field-grown and GH-grown fruit: generally field fruit exhibited higher injuries (with CI approaching 4) than GH fruit (CI approaching 3) (Figures 1A,B).

### Assessment of water loss due to chilling stress

**Figure 2:**
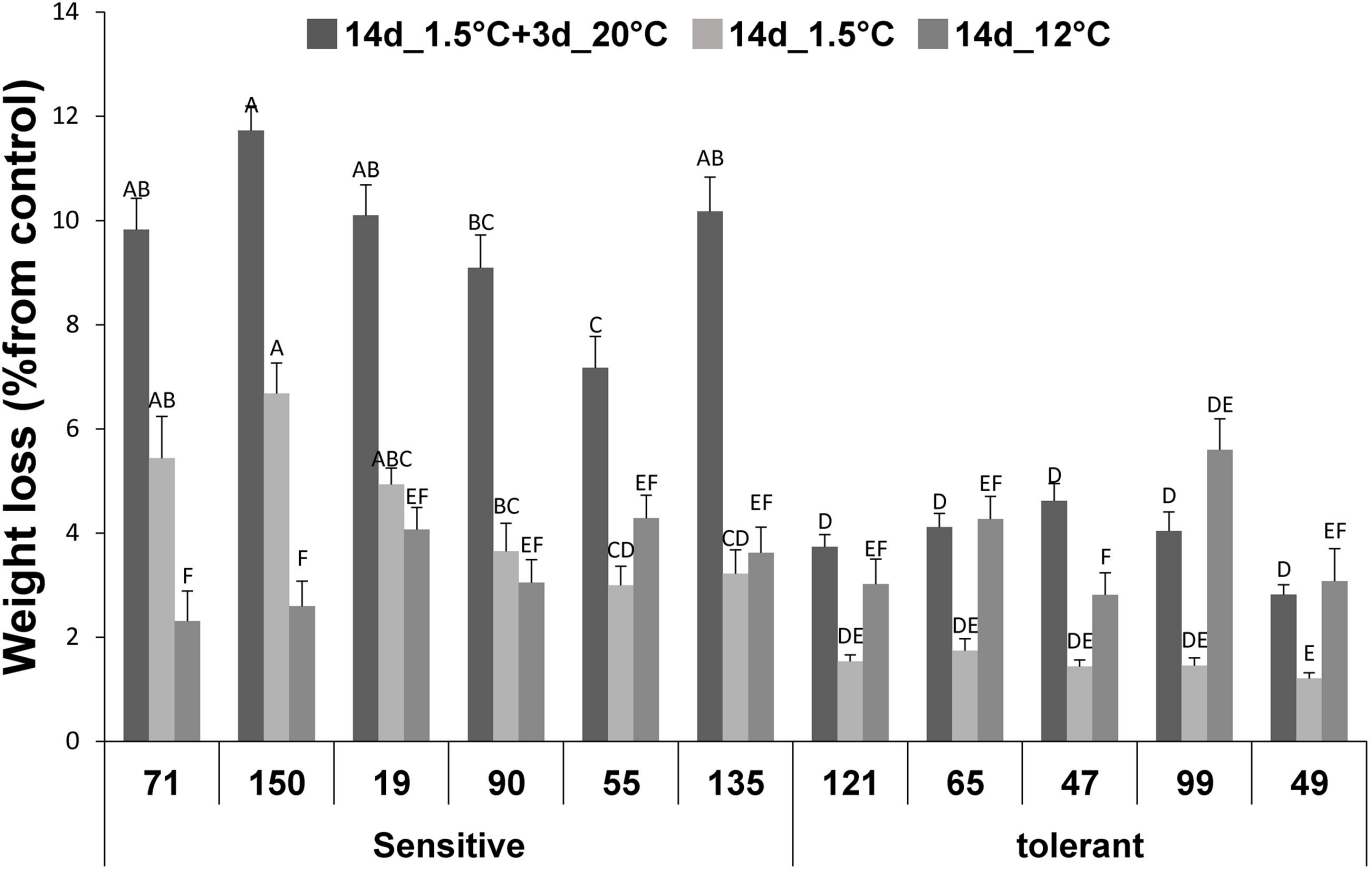
Weight loss values after storage at chilling of fruits from different tomato RILs. Fruits at the MG ripening stage were harvested and stored at 1.5°C or at 12°C (control). The fresh WL was determined immediately after 14 of storage. The changes in WL are relative to the fruit weight measured before storage as described in Materials and Methods. The results represent an average of 20–30 fruits for each RIL. Vertical bars are standard error means and different letters indicate a significant difference (*p* < 0.05).

Following 14-d storage at 1.5°C, water loss in the 5 chilling-tolerant RILs examined was around 1.5–2% reduction in FW, compared to their initial FW (Figure 2). Under the same conditions, the water loss was significantly higher in the 6 chilling-sensitive RILs examined, averaging 3–6% reduction in FW (Figure 2). For all the 11 RILs examined, 3 days of subsequent storage of the fruit at 20°C accelerated the reduction in FW and development of shriveling, indicative of further water loss by about two-fold (Figure 2). Under the conditions of cold storage + 3 days at 20°C, the difference in water loss was significant between the chilling-tolerant and chilling-sensitive RILs, with the latter exhibiting 2–3-fold higher water loss. It should be noted that, fruit that were stored under the control conditions (12°C) exhibited similar loss in FW (2–4%) in both the tolerant and sensitive RILs (Figure 2).

### Determination of the relationship between chlorophyll fluorescence and fruit chilling tolerance

**Figure 3:**
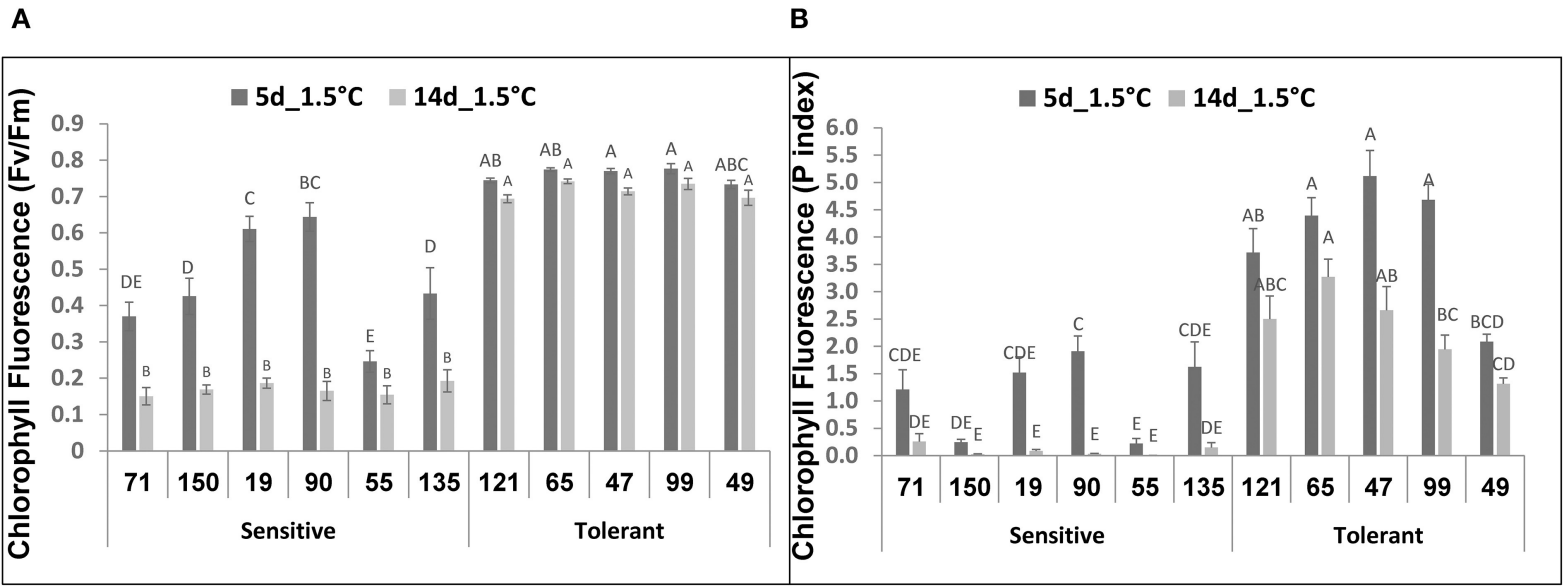
*Chlorophyll Fluorescence* values after storage at chilling temperature of fruits from different tomato RILs. Fruits at the MG ripening stage were harvested and immediately stored for 14 days at 1.5°C. The *Fv/Fm* **(A)** and P Index **(B)** values were determined after 5 and 14 days of storage as described in Materials and Methods. The results represent an average of 15-30 fruits for each RIL. Vertical bars are standard error means and different letters (A–E) indicate a significant difference (*p* < 0.05).

Following 5-d storage at 1.5°C, the *Fv/Fm* values for the chilling-tolerant fruit were about 0.7–0.8, which was close to the value for a non-damaged photosynthetic system (Figure 3A). In contrast, *Fv/Fm* values for the chilling-sensitive fruit were significantly lower, 0.25–0.65 (Figure 3A). Similarly, measurements of *Fv/Fm* values after 14 days at 1.5°C indicated high values of 0.7–0.75 for the 5 chilling-tolerant RILs and low values of 0.15 – 0.2 for the 6 chilling-sensitive RILs (Figure 3A). In control experiments, where fruit were stored at 12°C, the *Fv/Fm* values for both the chilling-tolerant and chilling-sensitive RILs were in the range of 0.75–0.85, (Supplementary Figure 3). In agreement with the *Fv/Fm* values, the photosynthesis *performance index* (*P. Index*) under 1.5°C was significantly higher for most (4 out of 5) of the chilling-tolerant RILs, compared with the chilling-sensitive RILs (Figure 3B). It should be noted that, when the *P. Index* was measured immediately after harvest, all RILs showed similar values, in the range of 5.0–6.7. These values were reduced by about 30% when measured after 14 days of storage at 12°C, with values in the range of 3.6–5.5 (Supplementary Figure 4).

### Determination of fruit proline content following low-temperature storage

**Figure 4:**
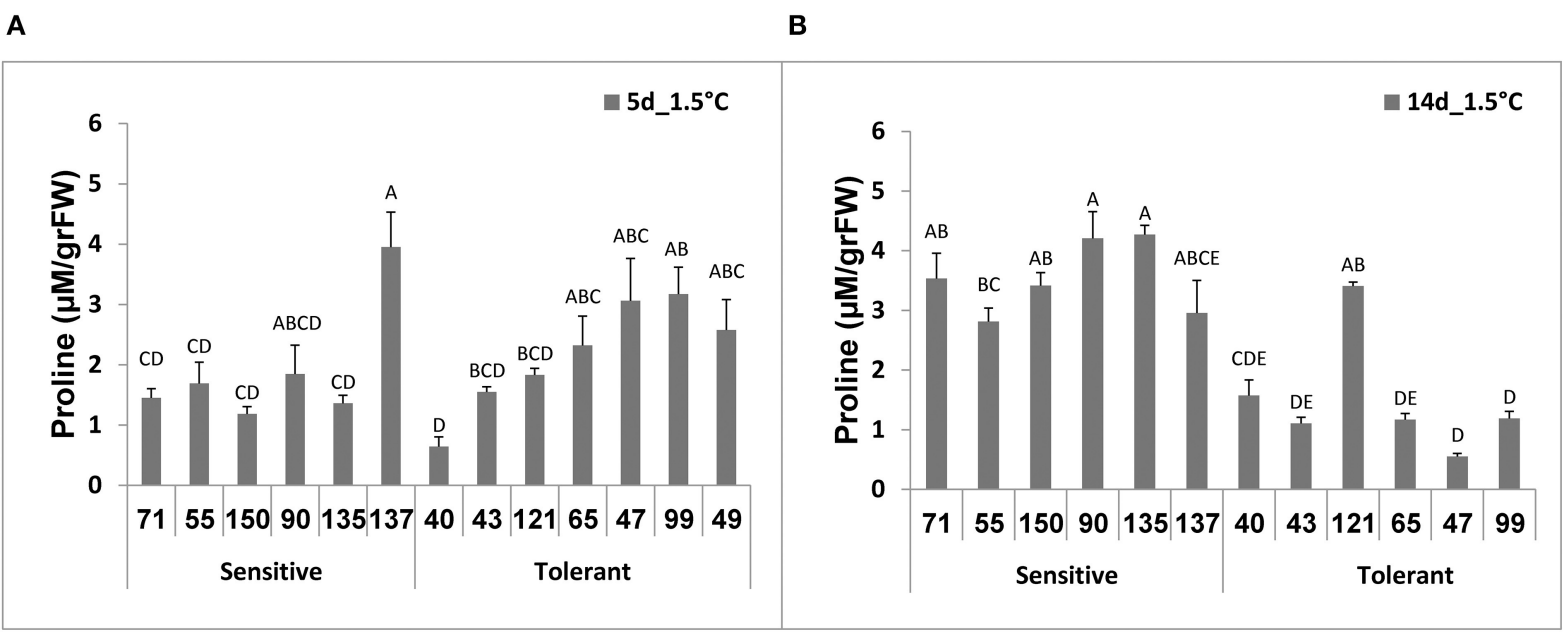
Proline content measured after cold storage of fruits from different tomato RILs. Fruits at the MG ripening stage were harvested and immediately stored for 14 days at a chilling temperature of 1.5°C. Proline content was determined after 5 **(A)** and 14 days **(B)** of storage as described in Materials and Methods. The results represent an average for 3–8 fruits for each RIL, each repeat consisting of 3–5 fruits. Vertical bars are standard error means and different letters (A–E) indicate a significant difference (*p* < 0.05).

Following 5 days at 1.5°C storage, there were no significant differences between proline contents of the chilling-tolerant and chilling-sensitive RILs (Figure 4A). Following 14 days at 1.5°C storage, however, while fruit proline content of the chilling-tolerant RILs (all except RIL40 and RIL121) had declined, the proline content of the chilling-sensitive RILs was elevated (Figure 4B). In general, after 14 days at 1.5°C, the proline content in the tolerant RILs was lower than that in the sensitive RILs (Figure 4); while the proline level in the tolerant RILs ranged from 0.5 to 1.5 μM/gr FW, that of the sensitive RILs ranged from 2.7 to .2 μM/gr FW.

### Determination of relationships between chilling tolerance and antioxidant activity and ascorbic acid

**Figure 5:**
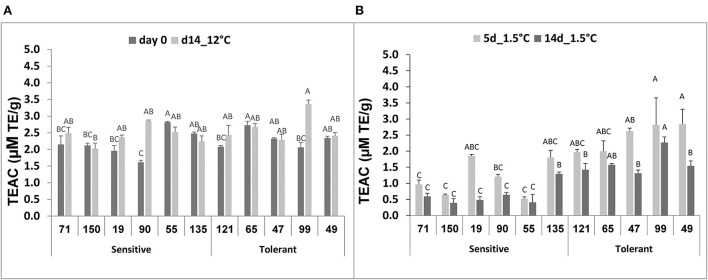
Total antioxidant activity before and after storage of fruits from different tomato RILs. Fruits at the MG ripening stage were harvested and stored at an optimal temperature of 12°C and a chilling temperature of 1.5°C. The total antioxidant activity was determined immediately after harvest and 14 days at 12°C **(A)** and 5 and 14 days at 1.5°C **(B)** using the TEAC method described in Materials and Methods. The results represent an average of 3–8 repeats for each RIL, and each repeat consists of 3–5 fruits. Vertical bars are standard error means and different letters indicate a significant difference (*p* < 0.05).

No significant difference was observed among the selected RILs when antioxidant activity was measured in fruit immediately after harvest or after 14-d storage at 12°C (Figure 5A). Following storage at 1.5°C, however, a general decline in antioxidant activity was observed in all RILs, and after 14-d chilling storage, the antioxidant activity was significantly lower in the chilling-sensitive RILs compared to the chilling-tolerant RILs (Figure 5B).

**Figure 6:**
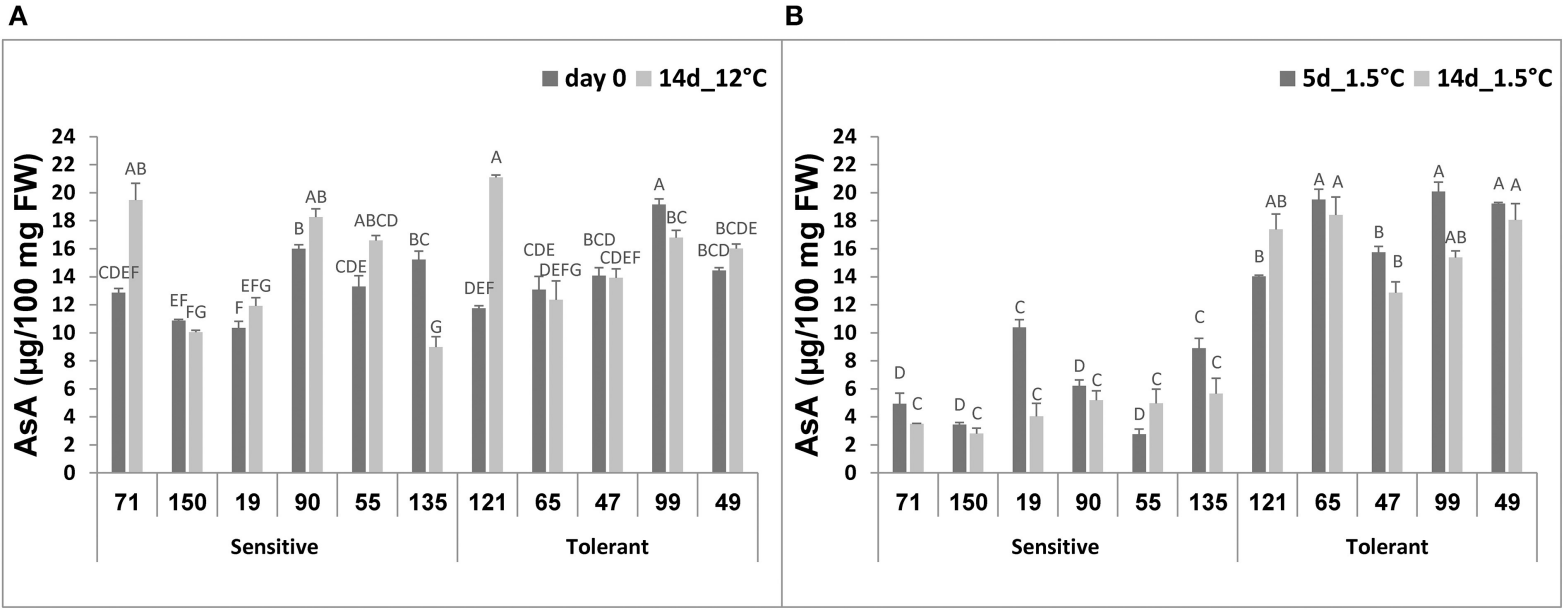
Ascorbic acid concentration before and after storage of fruits from different tomato RILs. Fruits at the MG ripening stage were harvested and stored at an optimal temperature of 12°C and a chilling temperature of 1.5°C. The AsA content was determined immediately after harvest and 14 days at 12°C **(A)** and after 5 and 14 days at 1.5°C **(B)** storage as described in Materials and Methods. The results represent an average for 3–6 repeats for each RIL, and each repeat consists of 3–5 fruits. Vertical bars are standard error means and different letters (A–G) indicate a significant difference (*p* < 0.05).

Ascorbic acid (AsA) content was similar in all selected RILs, immediately after harvest or following 14-d storage at 12°C, and it ranged from 10 to 20 μg/100 mg FW (Figure 6A). After 5-d storage at 1.5°C, the level of AsA in the chilling-tolerant RILs ranged from 15 to 20 μg/100 mg FW, and it did not change following 14-d storage at 1.5°C (Figure 6B). In contrast, the level of AsA in the chilling-sensitive RILs ranged from 3 to 10 μg/100 mg FW after 5-d storage at 1.5°C, and it further reduced (in all but RIL55) to 3–5 μg/100 mg FW following 14-d storage at 1.5° (Figure 6B). The difference in AsA content under chilling stress was significant between the chilling-tolerant and chilling-sensitive RILs.

### Determination of the expression of CBF cold-responsive gene in fruit stored under low temperature

**Figure 7:**
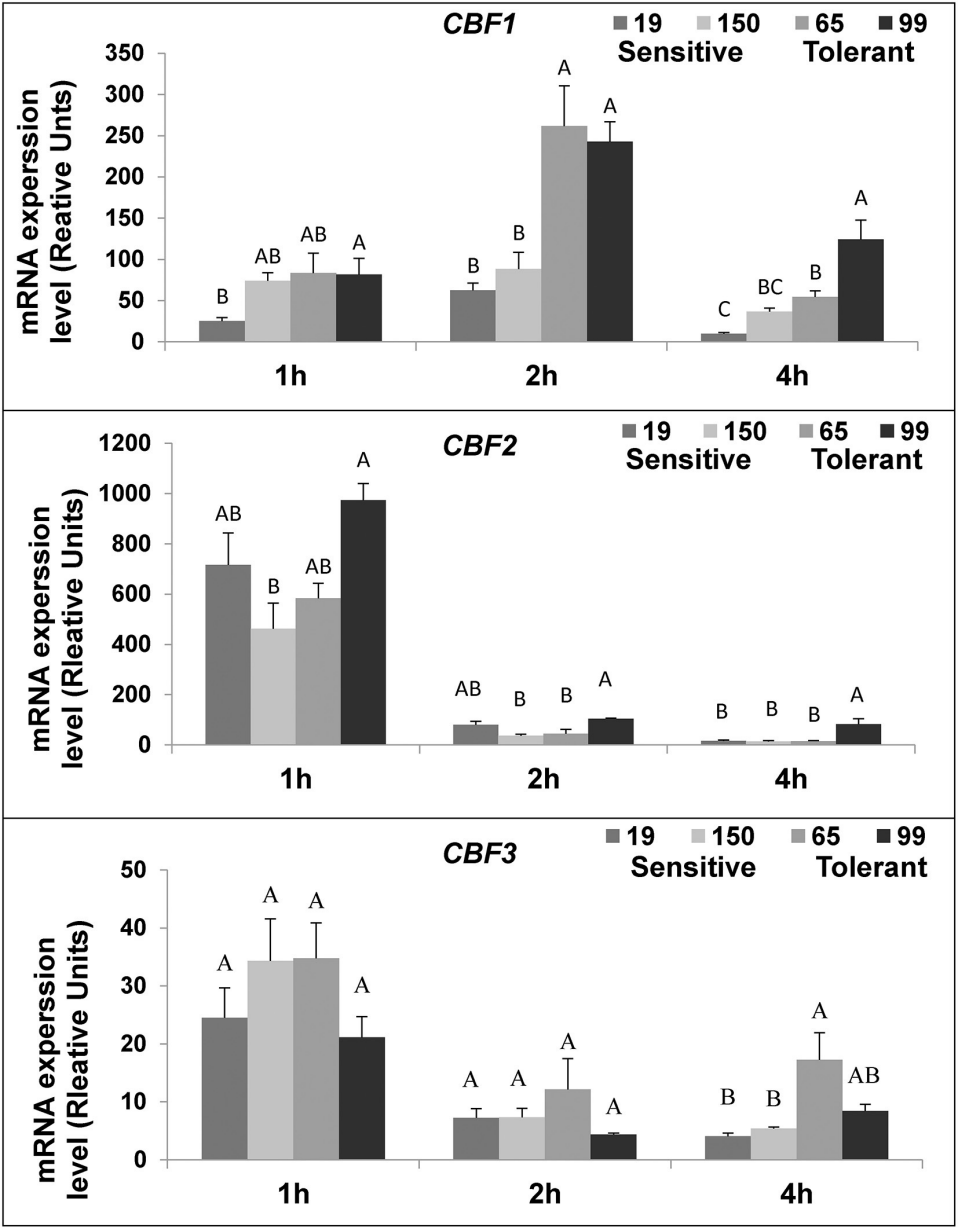
*SlCBF 1-3* genes expression after cold storage of fruits from different tomato RILs. Fruits at the MG ripening stage were harvested and immediately stored at 1.5°C. The *SlCBF* 1-3 expression was determined by qPCR after one, two, and four h, as described in Materials and Methods. Shown here are the results for cold-sensitive RIL 19 and 150 and cold-tolerant RIL 65 and 99. The results represent an average for 6-9 repeats. Each repeat consists of tissue samples from 5 fruits. Different letters (A-C) indicate a significant difference within each time point (*p* > 0.05); vertical bars are the standard error mean.

The expression of the three tomato *C-repeat Binding Factor* (*CBF*) transcriptional genes (*CBF 1-3*) was measured in fruit of two cold-tolerant (RIL65 and RIL 99) and two cold-sensitive RILs (RIL19 and RIL150) during the first few hours of cold storage. The chilling temperature triggered *CBF* gene expression during the first hour of exposure, compared to the initial expression level before chilling treatment (Figure 7). Differences, however, were observed among the three genes in their expression levels and kinetic patterns: while *CBF2* and *CBF3* expressions peaked in 1-hand then reduced, the *CBF1* expression peaked in 2-h (Figure 7). The expression level of *CBF1* was generally higher in the cold-tolerant compared to the cold-sensitive RILs (Figure 7).

## Discussion

An important step before marketing many fruit and vegetables is storage at low temperatures to prolong their marketing period and increase the total value. Many commercial fruit and vegetables, however, exhibit limitations as to their ability to withstand low temperatures during postharvest storage. While genetic variation exists in the ability to endure postharvest storage conditions, most cultivated species have lost such abilities due to genetic bottlenecks during their domestication and early breeding activities (Hu et al., 2020; Wang et al., 2020; Yolcu et al., 2020). In tomato, extensive genetic variation for abiotic stress tolerance exists within the related wild species (Boyer, 1982; Dwivedi et al., 2016), which could be exploited in breeding programs to enhance stress resiliency of modern cultivars (Von Wettberg et al., 2020; Yolcu et al., 2020). In the current study, we investigated the presence of variation in postharvest fruit chilling tolerance in a tomato RIL population, derived from a cross between a chilling-sensitive tomato breeding line (NC EBR1) and a chilling-tolerant accession (LA 2093) of the tomato wild species *S. pimpinellifolium*.

In the field experiment we observed significant variation in damages (e.g., tissue collapse) caused by storing fruit under chilling conditions, suggesting the presence of variation in fruit chilling tolerance in the RILs (Figure 1A). Similar observations were made when fruit from a select number of RILs grown under GH conditions were stored under chilling temperatures (Figure 1B), confirming the presence of genetic factors determining fruit response to low temperatures. The observation of generally higher *CI* scores for fruit collected from the field-grown plants, compared to fruit collected from the GH-grown plants, indicates the importance of pre-harvest plant growing conditions on fruit chilling resiliency postharvest. Among other factors, in our experiments, the RILs experienced higher temperatures under field conditions. Similar results were reported previously in tomatoes and other crops as to the influence of plant growing conditions and postharvest fruit chilling tolerance (Wang, 1997; Arah et al., 2015). It should be noted that, in our field experiment, no significant difference in *CI* scores was observed for the different harvest dates.

The presence of variation for cold tolerance during different stages of plant development, including seed germination and vegetative growth, was previously reported in different tomato wild accessions (Foolad and Lin, 2000, 2001a,b; Cao et al., 2015). Several studies also examined genetic bases of cold tolerance in tomato, and reported the presence of useful genetic variation that could be utilized in breeding programs (Foolad et al., 1998; Venema et al., 2005; Kazmi et al., 2012; Liu et al., 2016; Dinh et al., 2019). During the past few years, we have established a cooperative project to identify desirable sources of chilling tolerance in tomato fruit during postharvest storage, determine the genetic and physiological bases of the trait, and to develop new tomato germplasm with fruit chilling tolerance. In the present study, we determined the presence of genetic variation in fruit chilling tolerance in a tomato RIL population. This finding is important considering that recently we developed a highly saturated genetic map of this population with >140,000 genetic markers (Gonda et al., 2019), which could be utilized to investigate the molecular and physiological genetic basis of tomato fruit chilling tolerance in this population. The results of the current study will also facilitate determination of any correlation that may exist between postharvest fruit chilling tolerance and cold tolerance during plant growth and development. Such studies are currently underway in our programs.

We examined correlations between fruit visual damages caused by the chilling temperature during storage and several physiological, biochemical and molecular parameters that were previously shown to correlate with cold-stress-induced-responses (Sevillano et al., 2009). For example, the chlorophyll fluorescence *Fv/Fm* is a well-known parameter to assess the PSII efficiency in plants grown under various abiotic stresses (Walker et al., 1990; Mishra et al., 2014; Perez-Bueno et al., 2019). In our studies, we observed a clear reduction in *Fv/Fm* parameters under chilling stress in those RILs that exhibited fruit chilling sensitivity as determined by their visual symptoms (Figure 3A). In addition, a reduction in the level of the photosynthesis *P. index* was found to correspond well with fruit chilling sensitivity (Figure 3B). Previously, *P. Index* was identified as a reliable and sensitive parameter of plant homeostasis and abiotic stress tolerance, including tolerance to dark and chilling stresses (Clark et al., 2000; Strasser et al., 2004; Strauss et al., 2006; Zivcak et al., 2008; Kalaji et al., 2011; Ceusters et al., 2019). The overall results suggest that *Fv/Fm* and *P. Index* might be helpful parameters for fast, early, and sensitive screening of tomato fruit for their tolerance to postharvest chilling conditions, provided that experimental conditions are standardized for reliable application of these parameters.

We also determined relationships between fruit chilling tolerance during storage and two tissue antioxidant systems, namely the total antioxidant activity and the level of ascorbic acid (AsA, vitamin C). It should be noted that, before the cold storage treatment there were no significant differences in the two-antioxidant systems between the cold-tolerant and cold-sensitive RILs. Under the chilling stress conditions, the activities of both systems significantly reduced in the cold-sensitive RILs, whereas the levels of both systems were either not reduced or reduced insignificantly in fruit of the “cold-tolerant” RILs (Figures 5, 6). These results are in general agreement with previous studies where correlations were observed between the degree of tomato plant sensitivity/tolerance to chilling stress and the level/activity of different components of antioxidant systems (Wang et al., 2005; Sevillano et al., 2009; Lukatkin et al., 2012). Other studies reported positive correlations between the level of AsA and post-harvest chilling tolerance in various plant species, including tomato (Stevens et al., 2008; Wang et al., 2012; El Airaj et al., 2013; Qin et al., 2015). It is known that AsA is a major component of plants' antioxidant systems, participating in neutralizing harmful effects of ROS (Smirnoff, 2018). Previous studies have shown that, in plants exposed to cold stress, the level of ROS increased in the cell with destructive consequences on various cellular components, if ROS was not neutralized (Prasad, 1996; Suzuki and Mittler, 2006). In general, low temperature exposure results in inefficient PSII, leading to higher generation of ROS (Foyer et al., 1994; Wise, 1995). Cold storage of fresh produce in the dark could also result in the elevation of ROS, leading to physical damages (Sevillano et al., 2009) such as disturbance of membrane lipids (Marangoni et al., 1996; Kratsch and Wise, 2000). Postharvest chilling has also been shown to induce oxidative stress in tomato fruit (Malacrida et al., 2006; Biswas et al., 2017). The overall results from this and previous studies suggest the importance of a strong antioxidant system to minimize damages in fruits stored under chilling temperatures. A higher proline content, generally observed in the cold sensitive RILs (Figure 4), may be linked to a less active antioxidative system. In general, tissues in which the antioxidant system is less active may experience elevated chilling-induced damages, which in turn may lead to enhanced proline accumulation for better protection. Accumulation of proline in plant cells has been observed to occur in response to abiotic stresses, including chilling stress, and suggested to act as an osmolyte, a ROS scavenger, and a molecular chaperone stabilizing the structure of proteins, thereby protecting cells from damages caused by the stress (Verbruggen and Hermans, 2008; Szabados and Savoure, 2010; Krasensky and Jonak, 2012).

The activation of defense mechanisms in response to cold stress is under complex regulatory networks, involving initiation of gene expression and subsequent tolerance mechanisms (Knight and Knight, 2012; Shi et al., 2018; Ding et al., 2019). Primary sensors for cold stress responses have been identified and localized in the membrane, which are activated early on upon exposure to the stress (Ma et al., 2015; Liu et al., 2017; Yuan et al., 2018). A central signaling pathway for the primary cold response and acclimation is the activation of ICE1-CBF/DREB-COR transcriptional networks in the nucleus (Thomashow, 1999; Park et al., 2015; Shi et al., 2018; Ding et al., 2019); CBFs activate transcription of various cold responsive genes, involved mainly in downstream cellular signal transduction and metabolic processes required for acclimation to the stress (Guy et al., 1985; Thomashow, 1999; Chinnusamy et al., 2007). Previous studies had demonstrated that, in tomato, *SICBF1-3* genes were associated with cold acclimation and had a rapid expression response to low temperatures (Zhang et al., 2004; Albornoz et al., 2019). We have observed a higher expression of *CBF1* gene in the fruit of two cold-tolerant RILs, compared to two cold sensitive RILs, following 2-h exposure to the cold stress (Figure 7). Such higher gene expression may regulate early cold defense-related activating mechanisms, which enable better adaptation to the stress in the cold tolerant RILs. In contrast, expression of *CBF2* and *CBF3* genes did not differ between the cold-tolerant and cold-sensitive RILs after exposure to the stress. These results are in agreement with a previous study where the *CBF1* gene, but not *CBF2* and *CBF3*, was shown to be cold-inducible in tomato seedlings, with the highest expression measured 2-h following exposure to the stress (Zhang et al., 2004). In a previous study (Weiss and Egea-Cortines, 2009), whereas *CBF1* expression was not induced in postharvest tomato fruit following 2-h exposure to cold stress, it was induced by 2-fold in leaves under cold exposure, compared to the control. There might be different reasons for the observed differences between the two studies, including the use of MG fruit exposed to 1.5° cold stress in our studies, and the use of fruit in breaker stage exposed 6°C in Weiss and Egea-Cortines (2009) studies.

Zhang et al. (2004) reported that, while overexpression of the tomato *CBF1* gene in Arabidopsis induced expression of CBF-targeted genes and increased freezing tolerance, overexpression of *CBF1* in tomato did not increase freezing tolerance. Ironically, heterologous expression of the Arabidopsis *CBF1* gene in tomato resulted in elevated tolerance to chilling and oxidative stresses (Hsieh et al., 2002). The importance of the tomato *CBF1* gene in cold stress responses is further supported by the finding that its knockout reduced cold tolerance during vegetative stage (Li et al., 2018). We speculate that, high expression of *CBF1* transcriptional activator in tomato may have a role in cold acclimation of the fruit in addition to its function in escalating cold tolerance in vegetative tissues.

In conclusion, the tomato RIL population used in this study exhibited variation in fruit response to postharvest chilling stress, as manifested by various physiological, biochemical and molecular changes. Further studies are required to identify molecular components that differ between the cold-tolerant and cold-sensitive RILs. Transcriptomic, as well as, other “omic” studies comparing the cold-tolerant with the cold-sensitive RILs can facilitate the identification of central biological mechanisms, and central regulatory genes, determining post-harvest chilling tolerance, which would facilitate breeding tomatoes for improved postharvest storage at low temperatures.

## Data availability statement

The original contributions presented in the study are included in the article/Supplementary material, further inquiries can be directed to the corresponding authors.

## Author contributions

SD, AL, MF, and EF contributed to the conception and design of the study. SD did most of the experiments. EL and SA-T provided technical experimental assistance. SD and AL wrote the first draft of the manuscript, which was substantially improved by MF and EF. All authors contributed to manuscript revision, read, and approved the submitted version.

## Funding

This research was supported by the Research Grant Awards Nos. IS-5323-20C and IS-4783-14F from BARD. The United States–Israel Binational Agricultural Research and Development Fund to AL and MF and by grant no. 1812/18 from the Israel Science Foundation (ISF) to AL.

## Conflict of interest

The authors declare that the research was conducted in the absence of any commercial or financial relationships that could be construed as a potential conflict of interest.

## Publisher's note

All claims expressed in this article are solely those of the authors and do not necessarily represent those of their affiliated organizations, or those of the publisher, the editors and the reviewers. Any product that may be evaluated in this article, or claim that may be made by its manufacturer, is not guaranteed or endorsed by the publisher.
