# Supplementary material for: Physiological genetic variation in tomato fruit chilling tolerance during postharvest storage
Source: Front Plant Sci. 2022 Sep 8;13:991983. doi: 10.3389/fpls.2022.991983 (PMC9493348; doi:10.3389/fpls.2022.991983)
Supplement: Supplementary file 1 [file Data_Sheet_1.PDF]

Gene	Primer sequence
UBI3	(F) 5’ - GGTTAAGCTCGCTGTGTTGCAG - 3’
	(R) 5’ - AAACGTAGGTGAGCCCACAC - 3’
CBF1	(F) 5’ - GTGGATGAGGAGGCGATCTTT- 3’
	(R) 5’ - CACATTGAGGTGGAGGTAGCA - 3’
CBF2	(F) 5’ - GCAAATATGGCAGAAGGACTCA - 3’
	(R) 5’ - TAAGCGTGCACATCAGCTAATTC- 3’
CBF3	(F) 5’ - ATGGATGAGGAAGCGTTATTCTG - 3’
	(R) 5’ - TTCAGTACATTGAGGTGGAGGT- 3’

Table S1. Primers used for quantitative real-time PCR reactions. The letters F and R indicate forward (F) and reverse (R) primers of the strand gene sequence.

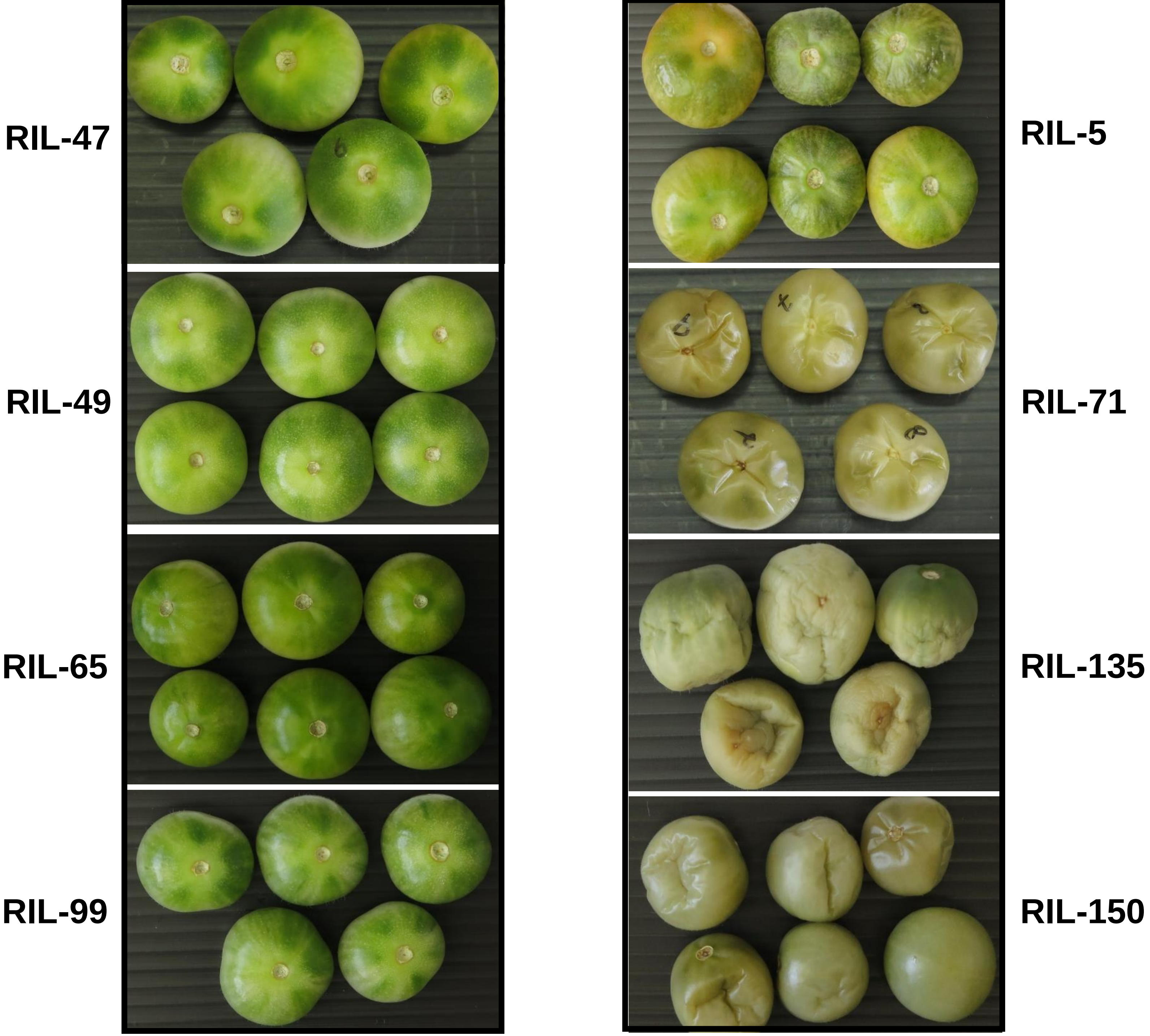

Figure S1. Development of surface chilling injuries in RIL fruit following postharvest cold storage. Examples for fruits from different tomato RILs, four tolerant RILs (left panel), and four sensitive RILs (right panel). Fruits at the MG ripening stage were harvested and immediately stored for 14 days at 1.5°C followed by three days at 20°C.

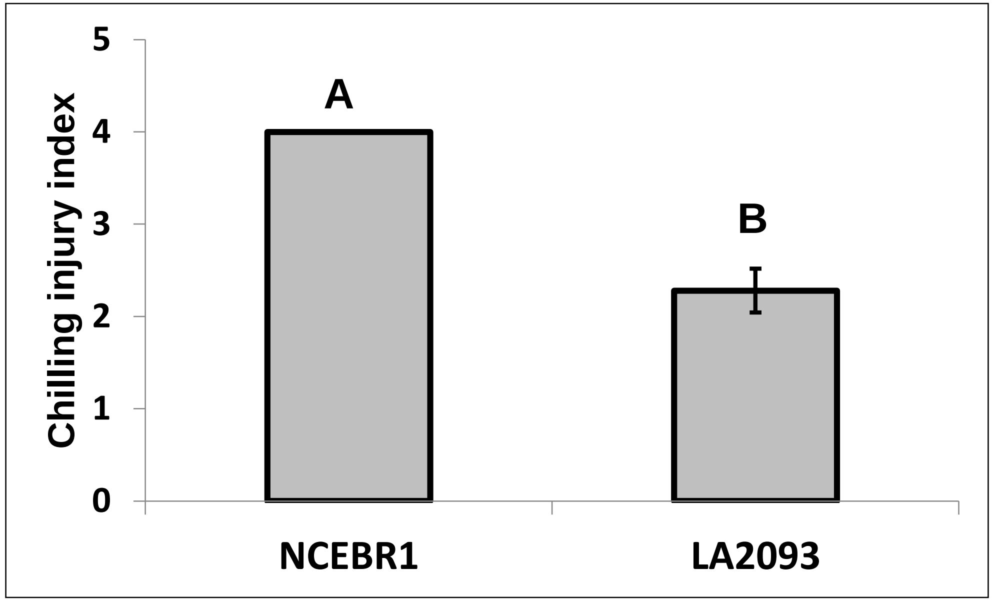

Figure S2. Susceptibility to chilling temperature storage of fruits from the parental lines, LA2093 and NCEBR1. Fruits at the MG ripening stage were harvested and immediately stored for 14 days at 1.5°C followed by 3 days at 20°C. Chilling injury scores were determined based on visual symptoms as described in Materials and Methods. Vertical bars are standard error means, and different letters indicate significant difference ($p < 0.05$).

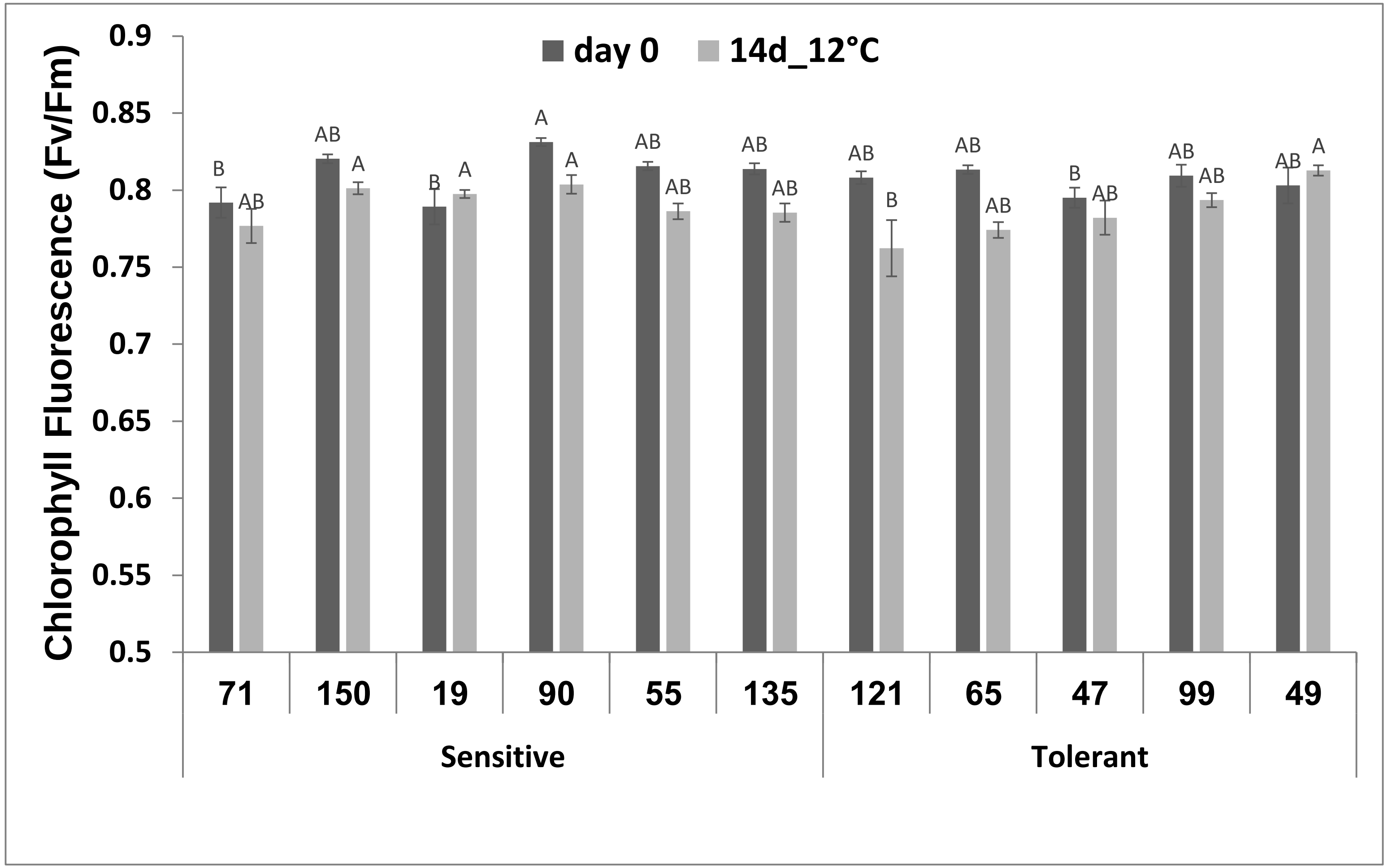

Figure S3. Fv/Fm values before storage and optimal temperature of fruits from different tomato RILs. Fruits at the MG ripening stage were harvested and immediately stored at an optimal temperature of 12°C. The Fv/Fm value was determined directly after harvest (day 0) and after 14 days of storage as described in Materials and Methods. The results represent an average of 15-30 fruits for each RIL. Vertical bars are standard error means, and different letters (A-E) indicate a significant difference ($p < 0.05$).

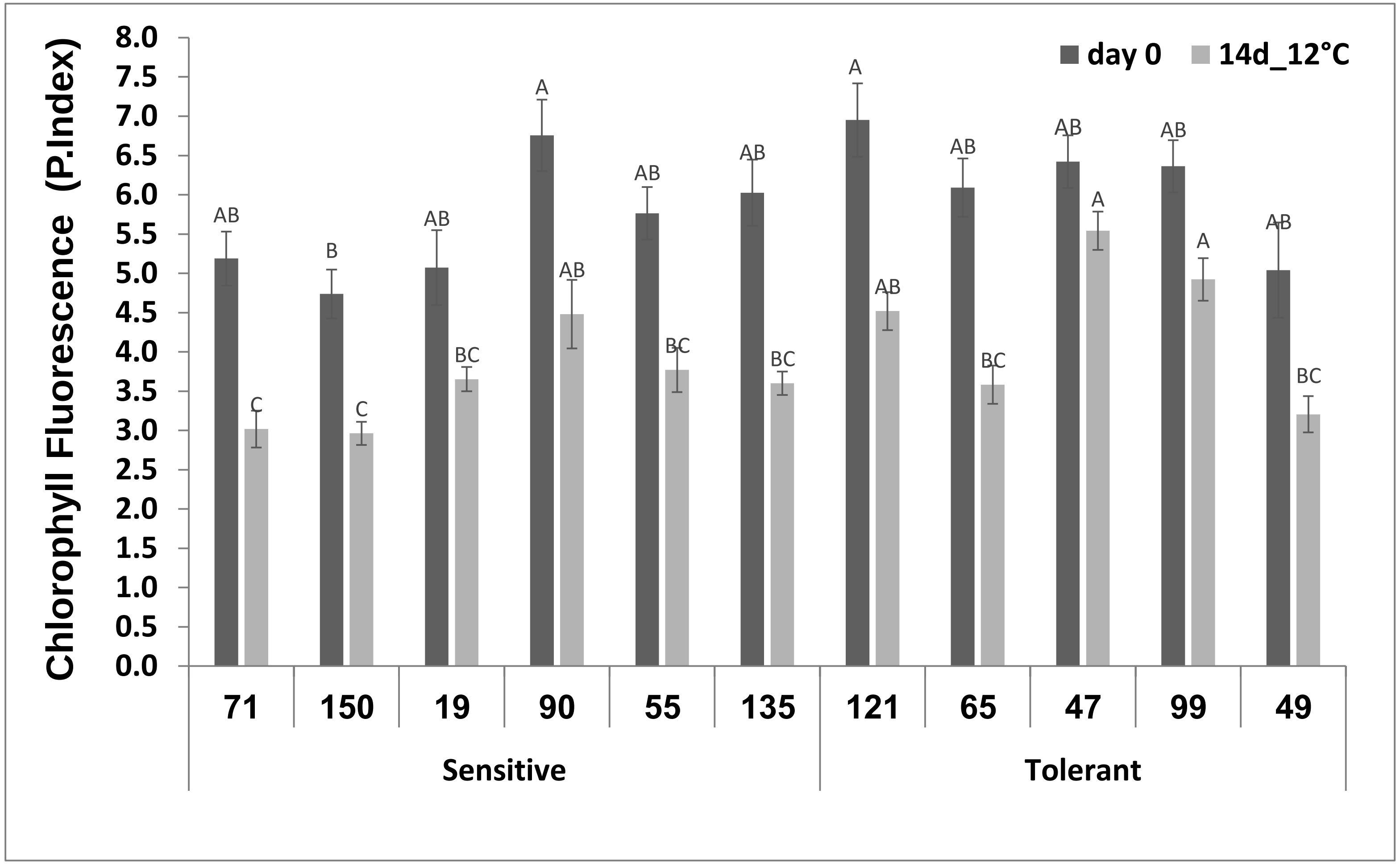

Figure S4. P. Index values before storage and at optimal temperature of fruits from different tomato RILs. Fruits at the MG ripening stage were harvested and immediately stored at optimal temperature of 12°C. The P. Index was determined directly after harvest (day 0) and 14 days of storage as described in Materials and Methods. The results represent an average of 15-30 fruits for each RIL. Vertical bars are standard error means, and different letters (A-E) indicate a significant difference ($p < 0.05$).
